# Complete Genome Sequence of *Serratia* Phage Muldoon

**DOI:** 10.1128/MRA.01418-19

**Published:** 2020-01-02

**Authors:** Soren Campbell, Cameron Atkison, Russell Moreland, Mei Liu, Jolene Ramsey, Justin Leavitt

**Affiliations:** a Center for Phage Technology, Texas A&M University, College Station, Texas, USA; Queens College

## Abstract

Serratia marcescens is a ubiquitous Gram-negative bacterium that is linked with emerging opportunistic infections. In this report, we describe the isolation and annotation of an S. marcescens myophage called Muldoon. Related to T4-like phages, such as Serratia phage PS2, Muldoon contains 257 predicted protein-coding genes and 4 tRNA genes.

## ANNOUNCEMENT

Serratia marcescens is an often pigmented Gram-negative member of the Enterobacteriaceae family (1). An increasing incidence of human disease caused by this pathogen is linked with multidrug resistance (2). Here, we describe the isolation, genome sequencing, and annotation of bacteriophage Muldoon, which targets S. marcescens.

Phage Muldoon was isolated from filtered (0.2-μm pore size) wastewater treatment plant samples collected in College Station, TX, by growth on Serratia marcescens D1 (catalog no. 8887172; Ward’s Science). The host was cultivated aerobically in LB (BD) at 30°C and 37°C. Muldoon was propagated by the soft-agar overlay method (3). Illumina TruSeq libraries were generated with a Nano low-throughput kit after DNA was purified, as described in the shotgun library preparation protocol by Summer (4), and phage Muldoon was sequenced on an Illumina MiSeq platform with paired-end 250-bp reads using v2 500-cycle chemistry. FastQC was used to control the quality of 565,076 total sequence reads (www.bioinformatics.babraham.ac.uk/projects/fastqc). The FastX Toolkit v0.0.14 (http://hannonlab.cshl.edu/fastx_toolkit/) was used for trimming before assembly with default parameters using SPAdes v.3.5.0 (5). The result was a contig with 69.2× coverage. To ensure that the complete termini were present, PCR products (forward, 5′-GTCACGATTTCCCTGCTATCT-3′; reverse, 5′-GCCGAATTTGCGTACGTTTAC-3′) amplified off the contig ends were Sanger sequenced. Structural annotation was carried out with GLIMMER v3.0 and MetaGeneAnnotator v1.0 for protein-coding genes and with ARAGORN v2.36 for tRNA genes (6–8). Rho-independent termination sites were annotated using TransTermHP v2.09 (9). Functional annotations were guided by results from InterProScan v5.33-72, BLAST v2.2.31, and TMHMM v2.0 analyses (10–12). BLAST searches were conducted with the NCBI nonredundant, UniProtKB Swiss-Prot, and UniProtKB TrEMBL databases at a maximum expectation value of 0.001 (13). Whole-genome comparisons were performed by the progressiveMauve v2.4.0 algorithm (14). Genomic terminus type was predicted with PhageTerm (15). All of the annotation tools listed above are in the Galaxy and Web Apollo instances hosted by the Center for Phage Technology at https://cpt.tamu.edu/galaxy-pub/ (16, 17). To determine morphology, Muldoon samples were negatively stained with 2% (wt/vol) uranyl acetate and viewed by transmission electron microscopy at the Texas A&M Microscopy and Imaging Center (18).

Myophage Muldoon has a 167,457-bp genome with a G+C content of 42%. With 257 predicted protein-coding genes and 4 tRNA genes, Muldoon has a 93% coding density. The genome was predicted to have permuted termini, indicating that this phage uses a T4-like packaging mechanism, and it was therefore reopened at the junction between its equivalents of the *rIIA* and *rIIB* genes to be syntenic with phage T4 (GenBank accession no. NC_000866). Unlike the T4 genome, Muldoon has no detectable introns. Phage Muldoon has its highest identity with *Serratia* phage PS2 (GenBank accession no. KJ025957), with 77.91% nucleotide identity and 253 similar proteins. Phage PS2 has a similarly large genome of 167,276 bp and has an identical number of tRNAs (19).

### Data availability.

The genome sequence and associated data for phage Muldoon were deposited under GenBank accession no. MN095771, BioProject accession no. PRJNA222858, SRA accession no. SRR8893603, and BioSample accession no. SAMN11414488.

## References

[1] Hejazi A, Falkiner FR 1997 Serratia marcescens. J Med Microbiol 46:903–912. doi:10.1099/00222615-46-11-903. 9368530

[2] Iguchi A, Nagaya Y, Pradel E, Ooka T, Ogura Y, Katsura K, Kurokawa K, Oshima K, Hattori M, Parkhill J, Sebaihia M, Coulthurst SJ, Gotoh N, Thomson NR, Ewbank JJ, Hayashi T 2014 Genome evolution and plasticity of *Serratia marcescens*, an important multidrug-resistant nosocomial pathogen. Genome Biol Evol 6:2096–2110. doi:10.1093/gbe/evu160. 25070509 PMC4231636

[3] Adams M 1959 Bacteriophages. Interscience Publishers, Inc, New York, NY.

[4] Summer EJ 2009 Preparation of a phage DNA fragment library for whole genome shotgun sequencing. Methods Mol Biol 502:27–46. doi:10.1007/978-1-60327-565-1_4. 19082550

[5] Bankevich A, Nurk S, Antipov D, Gurevich AA, Dvorkin M, Kulikov AS, Lesin VM, Nikolenko SI, Pham S, Prjibelski AD, Pyshkin AV, Sirotkin AV, Vyahhi N, Tesler G, Alekseyev MA, Pevzner PA 2012 SPAdes: a new genome assembly algorithm and its applications to single-cell sequencing. J Comput Biol 19:455–477. doi:10.1089/cmb.2012.0021. 22506599 PMC3342519

[6] Delcher AL, Harmon D, Kasif S, White O, Salzberg SL 1999 Improved microbial gene identification with GLIMMER. Nucleic Acids Res 27:4636–4641. doi:10.1093/nar/27.23.4636. 10556321 PMC148753

[7] Noguchi H, Taniguchi T, Itoh T 2008 MetaGeneAnnotator: detecting species-specific patterns of ribosomal binding site for precise gene prediction in anonymous prokaryotic and phage genomes. DNA Res 15:387–396. doi:10.1093/dnares/dsn027. 18940874 PMC2608843

[8] Laslett D, Canback B 2004 ARAGORN, a program to detect tRNA genes and tmRNA genes in nucleotide sequences. Nucleic Acids Res 32:11–16. doi:10.1093/nar/gkh152. 14704338 PMC373265

[9] Kingsford C, Ayanbule K, Salzberg S 2007 Rapid, accurate, computational discovery of rho-independent transcription terminators illuminates their relationship to DNA uptake. Genome Biol 8:R22. doi:10.1186/gb-2007-8-2-r22. 17313685 PMC1852404

[10] Jones P, Binns D, Chang HY, Fraser M, Li W, McAnulla C, McWilliam H, Maslen J, Mitchell A, Nuka G, Pesseat S, Quinn AF, Sangrador-Vegas A, Scheremetjew M, Yong SY, Lopez R, Hunter S 2014 InterProScan 5: genome-scale protein function classification. Bioinformatics 30:1236–1240. doi:10.1093/bioinformatics/btu031. 24451626 PMC3998142

[11] Camacho C, Coulouris G, Avagyan V, Ma N, Papadopoulos J, Bealer K, Madden TL 2009 BLAST+: architecture and applications. BMC Bioinformatics 10:421. doi:10.1186/1471-2105-10-421. 20003500 PMC2803857

[12] Krogh A, Larsson B, von Heijne G, Sonnhammer EL 2001 Predicting transmembrane protein topology with a hidden Markov model: application to complete genomes. J Mol Biol 305:567–580. doi:10.1006/jmbi.2000.4315. 11152613

[13] UniProt Consortium. 2019 UniProt: a worldwide hub of protein knowledge. Nucleic Acids Res 47:D506–D515. doi:10.1093/nar/gky1049. 30395287 PMC6323992

[14] Darling AE, Mau B, Perna NT 2010 progressiveMauve: multiple genome alignment with gene gain, loss and rearrangement. PLoS One 5:e11147. doi:10.1371/journal.pone.0011147. 20593022 PMC2892488

[15] Garneau JR, Depardieu F, Fortier LC, Bikard D, Monot M 2017 PhageTerm: a tool for fast and accurate determination of phage termini and packaging mechanism using next-generation sequencing data. Sci Rep 7:8292. doi:10.1038/s41598-017-07910-5. 28811656 PMC5557969

[16] Afgan E, Baker D, Batut B, van den Beek M, Bouvier D, Cech M, Chilton J, Clements D, Coraor N, Gruning BA, Guerler A, Hillman-Jackson J, Hiltemann S, Jalili V, Rasche H, Soranzo N, Goecks J, Taylor J, Nekrutenko A, Blankenberg D 2018 The Galaxy platform for accessible, reproducible and collaborative biomedical analyses: 2018 update. Nucleic Acids Res 46:W537–W544. doi:10.1093/nar/gky379. 29790989 PMC6030816

[17] Lee E, Helt GA, Reese JT, Munoz-Torres MC, Childers CP, Buels RM, Stein L, Holmes IH, Elsik CG, Lewis SE 2013 Web Apollo: a Web-based genomic annotation editing platform. Genome Biol 14:R93. doi:10.1186/gb-2013-14-8-r93. 24000942 PMC4053811

[18] Valentine RC, Shapiro BM, Stadtman ER 1968 Regulation of glutamine synthetase. XII. Electron microscopy of the enzyme from *Escherichia coli*. Biochemistry 7:2143–2152. doi:10.1021/bi00846a017. 4873173

[19] Teng T, Zhang G, Fan X, Zhang Z, Zhang L, Wu D, Chen S, Li Y, Jin J 2018 Complete genome sequence analysis of PS2, a novel T4-like bacteriophage that infects *Serratia marcescens* clinical isolates. Arch Virol 163:1997–2000. doi:10.1007/s00705-018-3803-0. 29574589

